# Phase III randomized trial of autologous cytokine-induced killer cell immunotherapy for newly diagnosed glioblastoma in korea

**DOI:** 10.18632/oncotarget.12273

**Published:** 2016-09-27

**Authors:** Doo-Sik Kong, Do-Hyun Nam, Shin-Hyuk Kang, Jae Won Lee, Jong-Hee Chang, Jeong-Hoon Kim, Young-Jin Lim, Young-Cho Koh, Yong-Gu Chung, Jae-Min Kim, Choong-Hyun Kim

**Affiliations:** ^1^ Department of Neurosurgery, Samsung Medical Center, Sungkyunkwan University School of Medicine, Seoul, Korea; ^2^ Department of Neurosurgery, College of Medicine, Korea University, Seoul, Korea; ^3^ Department of Statistics, Korea University, Seoul, Korea; ^4^ Department of Neurosurgery, Yonsei University College of Medicine, Seoul, Korea; ^5^ Department of Neurosurgery, Asan Medical Center, University of Ulsan College of Medicine, Seoul, Korea; ^6^ Department of Neurosurgery, Kyunghee University Hospital, Seoul, Korea; ^7^ Department of Neurosurgery, Konkuk University Medical Center, Seoul, Korea; ^8^ Department of Neurosurgery, Hanyang University Guri Hospital, Hanyang University College of Medicine, Guri, Korea

**Keywords:** immunotherapy, autologous cytokine-induced killer cell, glioblastoma

## Abstract

**Purpose:**

Adoptive cell immunotherapy involves an ex vivo expansion of autologous cytokine-induced killer (CIK) cells before their reinfusion into the host. We evaluated the efficacy and safety of CIK cell immunotherapy with radiotherapy-temozolomide (TMZ) for the treatment of newly diagnosed glioblastomas.

**Experimental design:**

In this multi-center, open-label, phase 3 study, we randomly assigned patients with newly diagnosed glioblastoma to receive CIK cell immunotherapy combined with standard TMZ chemoradiotherapy (CIK immunotherapy group) or standard TMZ chemoradiotherapy alone (control group). The efficacy endpoints were analyzed in the intention-to-treat set and in the per protocol set.

**Results:**

Between December 2008 and October 2012, a total of 180 patients were randomly assigned to the CIK immunotherapy (*n* = 91) or control group (*n* = 89. In the intention-to-treat analysis set, median PFS was 8.1 months (95% confidence interval (CI), 5.8 to 8.5 months) in the CIK immunotherapy group, as compared to 5.4 months (95% CI, 3.3 to 7.9 months) in the control group (one-sided log-rank, *p* = 0.0401). Overall survival did not differ significantly between two groups. Grade 3 or higher adverse events, health-related quality of life and performance status between two groups did not show a significant difference.

**Conclusions:**

The addition of CIK cells immunotherapy to standard chemoradiotherapy with TMZ improved PFS. However, the CIK immunotherapy group did not show evidence of a beneficial effect on overall survival.

## INTRODUCTION

Glioblastoma (GBM) is the most aggressive primary parenchymal brain tumor, which has a median survival of only 14.6-16 months. [1, 2] The current standard therapy is safe maximal resection followed by concurrent radiotherapy and chemotherapy with temozolomide (TMZ) and subsequent TMZ treatment. Despite current treatment regimens, there has been only modest improvement in survival. The dismal prognosis necessitates exploration of new or adjuvant treatment options to improve survival.

Over the past several centuries, whether the body's innate immune system could work to recognize and attack a malignant tumor has been a matter of debate. Immunotherapy was not considered an effective treatment option in brain tumors because of the blood brain barrier (BBB), as well as the absence of the lymphatic drainage system. [3] Cancer immunotherapy remains an available treatment option for certain cancer types. Adoptive cell immunotherapy relies on an ex vivo expansion of the autologous tumor-specific effector cells before their reinfusion into the host. [4] Adoptive cell therapy is a highly personalized cancer therapy that involves administration to the cancer-bearing host of immune cells with direct anticancer activity. Cytokine-induced killer (CIK) cells are major histocompatibility (MHC)-unrestricted cytotoxic lymphocytes that can be generated in vitro from peripheral blood mononuclear cells and cultured with the addition of interferon (IFN)-γ, interleukin (IL)-2, and CD3 monoclonal antibody (CD3mAb). [5] They have a high proliferative rate and anti-tumor activity, and can serve as an alternative cellular immunotherapy. [6] Recently, the application of CIK cells has evolved from experimental studies to phase II clinical studies. [3, 4, 6–11] Since the first clinical trial of adoptive immunotherapy using CIK cells in 1999, a growing number of trials have suggested that CIK therapy is associated with a significantly prolonged mean survival time and disease control rate. [12] Recent studies demonstrated that immunotherapy with CIK cells improved clinical outcome and promoted the quality of life (QoL) in cancer patients. [4, 5, 11, 13, 14] Wu, et al. reported the result of phase II study that chemotherapy plus CIK cells had potential benefits compared to chemotherapy alone in patients with advanced non-small cell lung cancer and autologous CIK cell transfusion had no obvious side-effects4. Recent literatures on CIK cells have demonstrated convincing evidence of the feasibility and very high safety profile in several clinical trials. [7, 10, 11, 15] Lee, et al. demonstrated adjuvant CIK immunotherapy improved survival for recurrent hepatocellular carcinoma in the phase III study15. Retrospective reviews regarding the adverse events by autologous CIK cells immunotherapy revealed that their adverse effects represented minor, self-limiting, and not serious, as most of the adverse events were resolved without additional management within 24 hours. [8, 9, 13, 15] However, there is no clinical evidence for adoptive immunotherapy using CIK cells to prolong the survival and promote the QoL in patients with GBM.

Here, we performed a multi-center, randomized, open-label phase III clinical trial to assess the efficacy and safety of autologous CIK cells administration for adoptive immunotherapy combined with the standard TMZ treatment in Korea with newly diagnosed GBMs.

## MATERIALS AND METHODS

### Study design

From December 2008 to October 2012, a prospective, randomized, phase III multi-center trial was conducted at 7 institutes in Korea. Patients were required to be registered before the start of concomitant chemo-radiotherapy with TMZ. Before randomization and after informed consent, an independent pathology review was done. Patients who met eligibility criteria were randomly assigned, in a 1 : 1 ratio, to receive either autologous CIK cells immunotherapy combined with standard TMZ chemoradiotherapy (CIK immunotherapy group) or standard TMZ chemoradiotherapy alone (control group). Random assignment was performed through a central telephone system using computer-generated permuted-block with a size of 4 or 6 and stratification according to each institute. Because this study was open label, we did not apply any masking procedures to study investigators or patients. The protocol was approved by the local institutional review board and scientific review boards prior to patient enrollment. This trial was also approved by the Ministry of Food and Drug Safety (MFDS, formerly known as the Korea Food and Drug Administration). Real-time monitoring of safety events was overseen by an independent data and safety monitoring board. The study was done, in accordance with the Declaration of Helsinki, the International Conference on Harmonisation note for good clinical practice (Topic E6, 1996), and applicable regulatory requirements. The data were collected by the sponsor, who vouched for data accuracy ( clinicaltrials.gov NCT 00807027).

### Standard TMZ chemoradiotherapy protocol

Standard TMZ treatment included concurrent radiotherapy (60 Gy administered as 2-Gy fractions 5 days per week) and chemotherapy with TMZ (75 mg per square meter of body-surface area per day for a maximum of 49 days). Maintenance treatment with TMZ began 4 weeks after the completion of radiotherapy at a starting dose of 150 mg per square meter for 5 consecutive days of a 28-day cycle, with an increase to 200 mg per square meter for subsequent cycles if treatment-related adverse events of grade 2 or higher were not noted. Maintenance TMZ was administered for up to 6 cycles.

### Generation of CIK cells and schedule of adoptive immunotherapy

For the autologous CIK immunotherapy group, peripheral blood ( > 120 ml) was collected from each patient at least 2 weeks before administration. This study used commercialized CIK cell agents manufactured in a GMP-certified central facility (Green Cross Cell Cop., Korea) following surgical procedure under strict control of quality and assurance, whereas the CIK cell preparation was performed by their own cultivating methods in the previous studies [15–17]. In brief, mononuclear cells were separated and cultured for 12-21 days with IL-2 and immobilized monoclonal antibody to CD3 at 372103. The CIK cell agent contained a total of 10^9^~2 x10^10^ cells in 200 mL of fluid consisting of 1% human albumin in normal saline. Immunotherapy group received CIK cell agent intravenously over 60 min, and were then observed for at least 30 min. They were scheduled to receive CIK cell agent 14 times (4 times at a frequency of once a week, followed by 4 times every 2 weeks, and finally 6 times every 4 weeks). CIK cells were administered at an outpatient clinic. Treatment could be delayed for maximum of 2 weeks under the following condition; 1, delay of patient's visit from his own schedule; 2, delay of treatment because of severe adverse effects; 3, in cases of contamination of CIK cells. Cytokines such as IFN, chemotherapeutic agents, other immunotherapeutic agents, hormonal therapy, and stem cell therapy were contraindicated during the study. The final cell products were assessed for viability by the dye-exclusion test, and checked twice for possible contamination by mycoplasma, sterility and endotoxin test.

### Eligibility

Patients enrolled in the study were aged 18 to 70 years; had a Karnofsky performance status (KPS) of at least 60; newly diagnosed GBM (World Health Organization [WHO] grade IV astrocytoma; with adequate hematologic, renal and hepatic function; and acceptable blood coagulation levels) as confirmed on central review. Patients were excluded if they received any prior therapy or had evidence of HIV-positive, immune-deficiency disease or auto-immune diseases such as rheumatoid arthritis, systemic lupus erythematosus, multiple sclerosis, or juvenile-type insulin-dependent diabetes mellitus, history of severe allergy, serious cardiac disease or a prior malignancy (except melanoma, cervical cancer *in situ*., and localized prostate cancer). Pregnant or breast feeding women were also excluded.

### Assessment of clinical responses

At baseline, all patients underwent a physical examination that included a neurologic assessment, complete blood counts, blood chemical analyses (including tests of renal and hepatic function), and tumor imaging with magnetic resonance imaging (MRI). Patients were invited to participate in a longitudinal evaluation of the net clinical benefits of the treatment with the use of a validated test for quality of life (the European Organization for Research and Treatment of Cancer (EORTC) quality of life questionnaire with a brain-cancer module (QLQ-C30). During radiotherapy, patients were assessed for adverse events weekly, and underwent weekly complete blood counts and blood chemical analyses. During the maintenance phase of treatment, patients underwent complete blood counts and blood chemical analyses on days 21 and 35 of each cycle.

Progression-free survival was assessed locally by investigators on the basis of enhanced MRI was performed approximately 4 weeks after chemoradiotherapy, then at 10, 22, 34 and 46 weeks after randomization, and every 3~12 months thereafter during the follow-up phase. In addition to investigator-assessed progression, two radiologists at an independent review facility analyzed all MRI scans. The independent reviewers were unaware of the study-group assignments, with read-only access to previous reviews until the final imaging data set was reviewed. Response was assessed with the use of serial measures of the product of the two largest cross-sectional diameters, and progression was defined as an increase in tumor size by at least 25% or the development of a new lesion based upon Macdonald criteria, integrating patient's general condition, infiltrative patterns on the T2/ FLAIR image, measurement of cerebral blood volume [18, 19]. Toxic effects were recorded and graded according to the National Cancer Institute Common Terminology Criteria for Adverse Events (CTCAE), version 3.0.

### Data analysis and statistical considerations

The primary endpoint was progression-free survival (PFS), and the goal of this study is to show that the addition of autologous CIK cells immunotherapy to a standard TMZ chemoradiotherapy would improve the PFS, as compared with the standard TMZ treatment alone. Secondary end points include overall survival (OS), objective responsive rate (ORR), disease control rate (DCR), Quality of life (QoL), KPS and assessment of adverse effects. PFS was calculated from the date of randomization either until disease progression was confirmed on the MR imaging or death from any causes. OS was calculated from the date of randomization until death from any cause. Objective response rate (OCR) was defined as either a partial or complete response and disease control rate (DCR) as partial, complete response or stable disease.

Sample size for the study was determined on the basis of PFS. Assuming a one-sided type I error of 0.05, a power of 80%, and a randomization ratio for 1:1 between two groups, 86 recurrence or death events were needed to detect a 20% difference (50% *vs*. 30%) in 12-month PFS. [16] When the potential “loss to follow-up” rate was set at 20%, 180 patients were required to record 86 recurrence or death events. The intention-to-treat (ITT) set was used to evaluate efficacy and included all randomized patients. The patients who completed treatment as planned were included in the per protocol set (PP set). Therapeutic outcomes were assessed based on the ITT set. Kaplan-Meier curves were generated for PFS and OS, and the log-rank test was used for group comparisons. A Cox's proportional hazard analysis was done to assess the treatment effect after adjustment for baseline characteristics.

Adverse events were compared between two groups by chi-square or Fisher's exact test. The log-rank test for the primary endpoint was one-sided, and all other statistical tests were two-sided. Statistical significance was set at *P* < .05. Statistical analysis was performed by statistician in the Department of Statistics at Korea university (Seoul, Korea) using SAS software version 9.2 (SAS Institute Inc., Cary, NC, USA).

### Study oversight

This trial was sponsored by Green Cross Cell company. We conducted the study at 7 sites. The steering committee provided oversight of the overall scientific integrity of the study. The protocol (available in supplementary file) was approved by the applicable independent ethics committees and institutional review boards. The study adhered to the principles of the Declaration of Helsinki and the Guidelines for Good Clinical Practice. The first draft of the manuscript was written by the first author with support from all coauthors; all authors reviewed and approved the manuscript. The data were collected by the sponsor and were analyzed by the statistician, 4th author (JW Lee). All the authors vouch for the completeness and accuracy of the data and confirm that the study was conducted according to the protocol.

## RESULTS

### Study patients

From December 2008 to October 2012, a total of 188 patients were screened from 7 institutes in Korea, 8 patients failed in the screening process, and 180 patients were randomized. 91 patients were randomized to the CIK immunotherapy group and 89 patients were randomized to the control group (Figure 2). 140 subjects were included in the per protocol set. The median age at the time of randomization was 55.0 and 54.0 years, with a range 19 to 69 and 23 to 68 years in each group. Males comprised 56.0% and 57.3% in each group, and all patients had KPS > 60 at the start of the study treatment (Table 1). Baseline characteristics except for the size of tumor were well balanced between the two treatment groups. Patients in the CIK immunotherapy group received the CIK cell agent containing 6.55 × 10^9^ cells per a treatment (Table 2).

**Figure 1:**
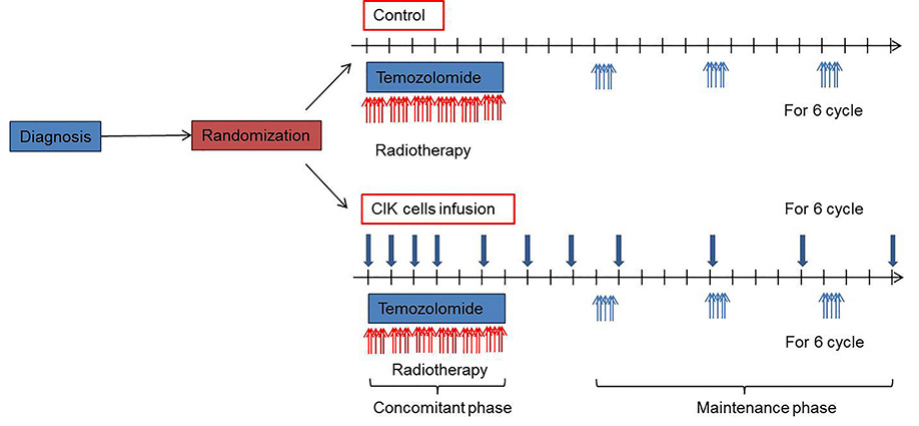
Treatment scheme

**Figure 2:**
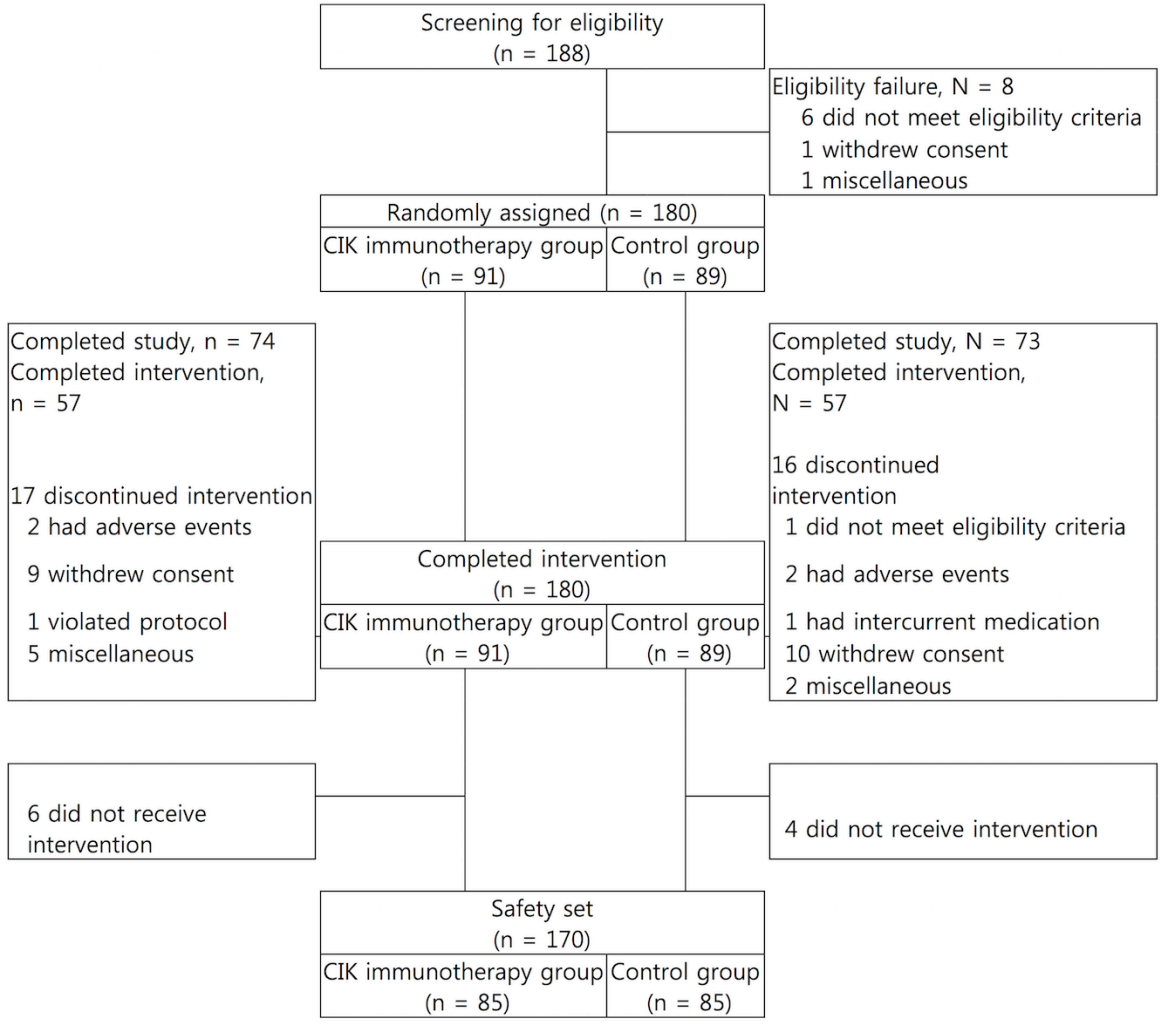
Paradigm of clinical trial for management of the newly diagnosed patients with glioblastoma

**Figure 3:**
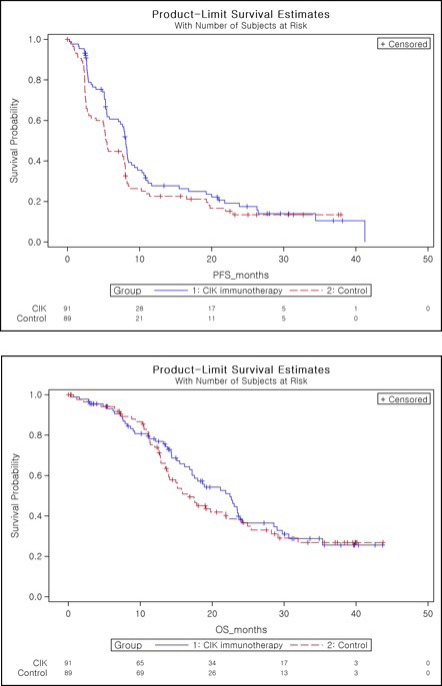
Kaplan-Meier plot of progression-free survival as assessed by investigator **A**. and overall survival **B**.

**Table 1:** Patient baseline characteristics and demographics (intention-to-treat set)

Patient Characteristics		CIK immunotherapy group	Control group
		*N* = 91	*N* = 89
		*n* (%)	*n* (%)
Age (years)	Mean ± SD	53.3 ± 10.8	52.8 ± 10.5
	Median	55.0	54.0
	Min, Max	19.0-69.0	23.0-68.0
Sex	Male	51 (56.0)	51(57.3)
	Female	40 (44.0)	38(42.7)
Extent of resection	Gross total resection	44 (48.4)	48(53.9)
	Subtotal resection	27 (29.7)	25(28.1)
	Partial resection	8 (8.8)	6(6.7)
	Biopsy only	12 (13.2)	10(11.2)
Karnofsky performance scale	Mean ± SD	84.4 ± 12.8	85.7 ± 13.1
	Median	90.0	90.0
	Min, Max	60.0-100.0	60.0-100.0
Global health status/ quality of life	n	86	85
	Mean ± SD	55.620 ± 22.340	54.314 ± 22.442
	Median	58	50
	Min, Max	8.0-100	0.0-100
Days from diagnosis to Baseline (Week 0)^2206^	Mean ± SD	21.6 ± 8.2	23.0 ± 9.5
	Median	21.0	22.0
	Min, Max	7.0-51.0	7.0-67.0
Days from surgery to Baseline (Week 0)^2302^	Mean ± SD	27.4 ± 8.1	28.7 ± 9.0
	Median	27.0	28.0
	Min, Max	10.0-55.0	9.0-69.0
Tumor size (cm)	Mean ± SD	5.7 ± 6.3	5.1 ± 10.4
	Median	4.2	1.4
	Min, Max	0.0-28.2	0.0-67.1

^2206^: The date of first diagnosis – Baseline + 1

^2302^: Surgery date – Baseline

**Table 2:** Summary of injected CIK cell agents (Safety set)

		Immunotherapy ( *n* = 85)
Total cell count (x10^9^)		
	Mean ± SD	6.55 ± 2.37
	Range	1.20-19.60
Cell viability (%)		
	Mean ± SD	97.81 ± 2.11
	Range	90.00-100.00
CD3^+^ cell (%)		
	Mean ± SD	98.63 ± 2.13
	Range	80.50-100.00
CD8^+^ cell (%)		
	Mean ± SD	83.67 ± 7.83
	Range	60.00-98.70
CD56^+^ cell (%)		
	Mean ± SD	28.23 ± 12.46
	Range	10.00-90.40
CD14^+^ cell (%)		
	Mean ± SD	0.04 ± 0.07
	Range	0.00-0.60
CD20^+^ cell (%)		
	Mean ± SD	0.15 ± 0.15
	Range	0.00-0.90
		n (%)
Injection times		
	0	6 (6.59)*
	1	1 (1.10)
	3-4	2 (2.20)
	6-10	25 (27.47)
	12-13	17 (18.68)
	14	40 (43.96)
Total injection no.	997	

*2 withdrew consent, 1 progression, 1 death, 1 deterioration, 1 miscellaneous

Note. There is no one who has been injected 2, 5, 11 times.

### Efficacy outcomes

In ITT set, median PFS was 8.1 months (95% confidence interval (CI), 5.8 to 8.5 months) in the CIK immunotherapy group, as compared to 5.4 months (95% CI, 3.3 to 7.9 months) in the control group (one-sided log-rank, *p* = 0.0401, hazard ratio 0.745 (90% CI, 0.564 to 0.985) (Table 3). Cox proportional hazard model with the baseline characteristics that might be clinically related with the efficacy endpoint was fitted with stepwise procedure. Treatment group was selected in ITT and PP set. The results showed the consistency in statistical significance (one-sided test for the treatment effect: ITT set: *p* = 0.0413, PP set: *p* = 0.0229, data not shown).

**Table 3:** Progression-free survival (PFS) in the intention-to-treat set

	CIK immunotherapy group (*N*= 91)	Control group (*N*= 89)
No. of events (Death or PD), n (%)	70 (76.9%)	71 (79.8%)
Median PFS [95% CI]	8.1 [5.8, 8.5]	5.4 [3.3, 7.9]
PFS rate (%)		
12 months (%) [95% CI]	28.3 [19.0, 38.3]	22.6 [14.3, 32.1]
18 months (%) [95% CI]	25.6 [16.7, 35.5]	21.2 [13.1, 30.6]
24 months (%) [95% CI]	18.4 [10.7, 27.9]	13.4 [6.8, 22.3]
*p*-value^1)^	0.0401^#^	
Hazard ratio [90% CI]^2)^	0.745 [0.564, 0.985]	

PD, progressive disease

* Progression-free survival=Diagnosis date of disease progression by MRI, date of death or date of off-study - Randomization date

^1)^ Results by one-sided Log-rank test

^2)^ Presented as the result of one-sided test

^#^ p-value < 0.05

The PFS rates with CIK immunotherapy group and control group were 28.3% and 22.6% at 1 year, and 18.4% and 13.4% at 2 years, respectively (Table 3). Considering the effect of accompanying steroid medication when interpreting tumor change on MRI, we identified that there was no significant difference in the steroid medication between both groups (Chi-square test, *p* = 0.60). In PP set, median PFS was 8.1 months (95% CI, 7.1 to 8.9 months) in the CIK immunotherapy group and 5.4 months (95% CI, 4.0 to 7.9 months) in the control group (one-sided log-rank, *p* = 0.0218, hazard ratio 0.693 (90% CI, 0.512 to 0.937)) (Supplementary Table 1). The median OS was 22.5 months (95% CI, 17.2 to 23.9 months) in the CIK immunotherapy group, as compared with 16.9 months (95% CI, 13.9 to 21.9 months) in the control group, However, our results did not show a statistically significant difference in OS between the two groups (two-sided log-rank test, *p* = 0.5237) (Table 4). Cox's proportional hazard model was fitted in the same way as PFS, and treatment group and tumor size were selected in both ITT set and PP set and the results showed the consistency in statistical significance (two-sided test for the treatment effect: ITT set: *p* = 0.5069, PP set: *p* = 0.4145, data not shown).

**Table 4:** Overall survival (OS) in the intention-to-treat set

	CIK immunotherapy group (*N*= 91)	Control group (*N*= 89)
Incidence rate (Death), n (%)	51 (56.04)	52 (58.43)
Median OS [95% CI]	22.47 [17.20, 23.85]	16.88 [13.91, 21.94]
OS rate (%)		
12 months (%) [95% CI]	78.22 [67.64, 85.69]	75.24 [64.26, 83.27]
18 months (%) [95% CI]	57.24 [45.27, 67.51]	45.08 [33.55, 55.93]
24 months (%) [95% CI]	38.21 [26.77, 49.54]	38.49 [27.22, 49.63]
p-value^1)^	0.5237	
Hazard ratio [90% CI]^2)^	0.693 [0.512, 0.937]	

*Overall Survival=Date of death or date of off-study - Randomization date

^1)^ Results by Log-rank test

^2)^ Presented as the result of one-sided test

There was no significant difference in the ORR including complete or partial response between groups (27.1% *vs*. 15.9%, *P* = 0.0783). However, a significant difference was found between groups in the DCR including complete response, partial response, and no change (82.4% *vs*. 63.4%, *P* = 0.0058) (Supplementary Table 3).

### Quality of life

All patients were required to complete the quality of life questionnaire. Global health status scores of the EORTC QLQ-C30 significantly worsened after both treatments, but there was no significant difference of change between the groups (*P* = 0.6409). In the detailed functional scales, there were no significant group-wise differences in physical, role, emotional, cognitive, and social functioning. In addition, symptom scales such as fatigue, nausea and vomiting, pain, dyspnea, insomnia, appetite loss, constipation, diarrhea, and financial difficulties did not show any significant difference between two groups. (Supplementary Table 4). On the KPS scale, there was no significant difference between two groups (Supplementary Table 5).

### Safety

Treatment-emergent adverse events (TEAEs) of any grade were reported in 84 (98.8%) of patients who received CIK immunotherapy group, and 83 (97.6%) of the control group (Table 5). The incidences of total and ≥ grade 3 were higher in the CIK immunotherapy group than the control group. However, there was no significant difference between groups (Supplementary Table 7). We did not find a significant difference in the rate of serious adverse events (41.2% *vs*. 36.5%, *P* = 0.5290), and in the rate of ≥ grade 3 adverse events (47.1% *vs*. 36.5%, *P* = 0.1616) (Supplementary Table 6). The incidences of total and ≥ grade 3 adverse drug reactions (ADRs) associated with CIK immunotherapy group was 16 patients (18.8%) and 3 patients (3.5%), respectively (7 events). Each case involved neutropenia in 5 events from 1 patient, pneumonia in 1 case, and acute renal failure in 1 case (Supplementary Table 8). No therapy was discontinued due to treatment-related complications. Cytokine storm or anaphylactic reactions were not observed during this clinical trial.

**Table 5:** Adverse events (AE, Safety set)

	CIK immunotherapy group^25C6^ (*N* = 85)												Control group^25C6^ (*N* = 85)					
	ADR						TEAE						TEAE					
	Total		Grade 3		Grade 4		Total		Grade 3		Grade 4		Total		Grade 3		Grade 4	
Preferred Term	n	(%)	n	(%)	n	(%)	n	(%)	n	(%)	n	(%)	n	(%)	n	(%)	n	(%)
overall incidence	16	(18.824)	3	(3.529)	0	(0.000)	84	(98.824)	39	(45.882)	10	(11.765)	83	(97.647)	27	(31.765)	5	(5.882)
Pyrexia	7	(8.235)	0	(0.000)	0	(0.000)	25	(29.412)	0	(0.000)	0	(0.000)	9	(10.588)	0	(0.000)	0	(0.000)
Chills	4	(4.706)	0	(0.000)	0	(0.000)	7	(8.235)	0	(0.000)	0	(0.000)	2	(2.353)	0	(0.000)	0	(0.000)
Fatigue	1	(1.177)	0	(0.000)	0	(0.000)	14	(16.471)	1	(1.177)	0	(0.000)	12	(14.118)	0	(0.000)	0	(0.000)
Pain	1	(1.177)	0	(0.000)	0	(0.000)	8	(9.412)	0	(0.000)	0	(0.000)	7	(8.235)	0	(0.000)	0	(0.000)
Urticaria	2	(2.353)	0	(0.000)	0	(0.000)	6	(7.059)	0	(0.000)	0	(0.000)	3	(3.529)	0	(0.000)	0	(0.000)
Erythema	1	(1.177)	0	(0.000)	0	(0.000)	3	(3.529)	0	(0.000)	0	(0.000)	3	(3.529)	0	(0.000)	0	(0.000)
Leukopenia	1	(1.177)	0	(0.000)	0	(0.000)	16	(18.824)	1	(1.177)	1	(1.177)	7	(8.235)	2	(2.353)	0	(0.000)
Neutropenia	1	(1.177)	1	(1.176)	0	(0.000)	4	(4.706)	3	(3.529)	0	(0.000)	3	(3.529)	0	(0.000)	0	(0.000)
Skin test positive	2	(2.353)	0	(0.000)	0	(0.000)	2	(2.353)	0	(0.000)	0	(0.000)	0	(0.000)	0	(0.000)	0	(0.000)
Pneumonia	1	(1.177)	1	(1.176)	0	(0.000)	5	(5.882)	2	(2.353)	1	(1.177)	5	(5.882)	1	(1.177)	1	(1.176)
Sepsis	1	(1.177)	0	(0.000)	0	(0.000)	3	(3.529)	2	(2.353)	0	(0.000)	0	(0.000)	0	(0.000)	0	(0.000)
Back pain	1	(1.177)	0	(0.000)	0	(0.000)	9	(10.588)	1	(1.177)	1	(1.177)	5	(5.882)	0	(0.000)	0	(0.000)
Myalgia	1	(1.177)	0	(0.000)	0	(0.000)	5	(5.882)	0	(0.000)	0	(0.000)	2	(2.353)	0	(0.000)	0	(0.000)
Diarrhoea	1	(1.177)	0	(0.000)	0	(0.000)	17	(20.000)	1	(1.177)	0	(0.000)	6	(7.059)	0	(0.000)	0	(0.000)
Hypersensitivity	1	(1.177)	0	(0.000)	0	(0.000)	1	(1.177)	0	(0.000)	0	(0.000)	4	(4.706)	0	(0.000)	0	(0.000)
Renal failure acute	1	(1.177)	1	(1.176)	0	(0.000)	1	(1.177)	1	(1.177)	0	(0.000)	0	(0.000)	0	(0.000)	0	(0.000)
Cough	1	(1.177)	0	(0.000)	0	(0.000)	9	(10.588)	0	(0.000)	0	(0.000)	7	(8.235)	0	(0.000)	0	(0.000)

Note. Listed are treatment-emergent adverse events (TEAEs) that were considered drug-related. Dictionary: MedDRA v16.0

25C6 overlapping count

## DISCUSSION

Recently, immunotherapy is an emerging candidate for alternative treatment options for a variety of cancers. [3, 14, 15, 17] Immunotherapy has recently evolved in the oncology field with therapeutic benefits and combinatorial regimen with cytotoxic agents is known to potentiate anti-tumor immune function.

CIK cells have anti-tumor activity *via* secretion of cytotoxic molecules and the production of multiple cytokines that regulate immune responses. [17] Adoptive cell therapy is a highly personalized cancer therapy that involves administration to the cancer-bearing host of immune cells with direct anticancer activity. Adoptive cell therapy provides a favorable microenvironment that better supports antitumor immunity. Such an immunotherapy would be more beneficial when residual tumors are minimal, such as in cases of postoperative adjuvant treatment. [16] This study was designed to determine whether adding autologous CIK cells immunotherapy to the first-line treatment for GBM would improve patient outcomes. We found that autologous CIK cells immunotherapy combined with standard chemoradiotherapy with TMZ was associated with a 2.7 months increase in median PFS in the newly diagnosed GBM. These results were comparable to the recently published clinical trials with Bevacizumab. [20, 21] In particular, autologous CIK immunotherapy group showed a higher DCR than the control group (82.4% *vs*. 63.4%, *P* = 0.0058). Furthermore, CIK immunotherapy adding to conventional temozolomide treatment did not induce additional deterioration of QoL, compared with the control group. It can be interpreted that maintenance of QoL could be a relevant treatment goal of CIK immunotherapy. Several studies have demonstrated the critical role of immunotherapy as an adjuvant treatment for residual or remnant caner. [6, 13] However, this was the first prospective randomized controlled study of CIK immunotherapy for newly diagnosed GBM. Our results raised the question of why anti-tumor activity of autologous CIK immunotherapy did not prolong the survival in this study, unlike previous phase II studies. BBB serves as a boundary that separates the peripheral circulation and the central nervous system, which inherently may prevent immune reaction in the brain. The efficacy of immunotherapy may depend upon the integrity of the BBB. As a result, immunotherapy may be more effective in advanced or recurrent cancer settings. Similar to CIK immunotherapy, randomized trials of bevacizumab or cilengitide added to standard chemoradiotherapy with TMZ in newly diagnosed GBM did not extend OS. [20-22] These results demonstrated that additional confirmative evidences are required before moving into definitive large-scale phase III study for newly diagnosed GBM. [22]

The limitation of this study is the lack of information about the molecular biomarkers in the GBM. Pre-informed stratification based upon the methylation status of O6-Methylguanine-DNA Methyltransferase (MGMT) gene promoter or isocitrate dehydrogenase 1 gene (IDH1) mutation must have enforced the power of the result in this study. In addition, the identification of surrogate immunologic biomarkers will accelerate the establishment of more successful immunotherapeutic strategies.

In summary, CIK immunotherapy for newly diagnosed GBM did not show statistically significant difference in OS but resulted in a significantly prolonged PFS, with relative maintenance of QoL and functional status. Significant adverse events related to treatment were not observed, and no adverse events appeared to be related to cytotoxicity. We did not identify a subgroup of patients who had a selected survival benefit from the CIK immunotherapy group. Further investigations of molecularly defined subgroups may uncover a predictive marker panel for autologous CIK immunotherapy, which would require additional prospective testing.

Provision of study materials or patients: Choong-Hyun Kim, Do-Hyun Nam, Shin-Hyuk Kang, Jong-Hee Chang, Jeong-Hoon Kim, Young-Jin Lim, Young-Cho Koh, Yong-Gu Chung, Jae-Min Kim

Collection and assembly of data: Choong-Hyun Kim, Doo-Sik Kong, Do-Hyun Nam, Shin-Hyuk Kang, Jong-Hee Chang, Jeong-Hoon Kim, Young-Jin Lim, Young-Cho Koh, Yong-Gu Chung, Jae-Min Kim

Data analysis and interpretation: Jae-Won Lee, Doo-Sik Kong, Do-Hyun Nam, Shin-Hyuk Kang, Choong-Hyun Kim

Manuscript writing: Doo-Sik Kong, Do-Hyun Nam, Choong-Hyun Kim

Final approval of manuscript: All authors

## SUPPLEMENTARY MATERIALS FIGURES AND TABLES



## References

[1] Gilbert MR, Wang M, Aldape KD, Stupp R, Hegi ME, Jaeckle KA, Armstrong TS, Wefel JS, Won M, Blumenthal DT, Mahajan A, Schultz CJ, Erridge S, Baumert B, Hopkins KI, Tzuk-Shina T, Brown PD, Chakravarti A, Curran WJ, Mehta MP (2013). Dose-dense temozolomide for newly diagnosed glioblastoma: a randomized phase III clinical trial. J Clin Oncol.

[2] Stupp R, van den Bent MJ, Hegi ME (2005). Optimal role of temozolomide in the treatment of malignant gliomas. Curr Neurol Neurosci Rep.

[3] McMahon EJ, Bailey SL, Miller SD (2006). CNS dendritic cells: critical participants in CNS inflammation?. Neurochem Int.

[4] Wu C, Jiang J, Shi L, Xu N (2008). Prospective study of chemotherapy in combination with cytokine-induced killer cells in patients suffering from advanced non-small cell lung cancer. Anticancer Res.

[5] Wang M, Cao JX, Pan JH, Liu YS, Xu BL, Li D, Zhang XY, Li JL, Liu JL, Wang HB, Wang ZX (2014). Adoptive immunotherapy of cytokine-induced killer cell therapy in the treatment of non-small cell lung cancer. PLoS One.

[6] Jakel CE, Hauser S, Rogenhofer S, Muller SC, Brossart P, Schmidt-Wolf IG (2012). Clinical studies applying cytokine-induced killer cells for the treatment of renal cell carcinoma. Clin Dev Immunol.

[7] Hontscha C, Borck Y, Zhou H, Messmer D, Schmidt-Wolf IG (2011). Clinical trials on CIK cells: first report of the international registry on CIK cells (IRCC). J Cancer Res Clin Oncol.

[8] Ma Y, Xu YC, Tang L, Zhang Z, Wang J, Wang HX (2012). Cytokine-induced killer (CIK) cell therapy for patients with hepatocellular carcinoma: efficacy and safety. Exp Hematol Oncol.

[9] Ma Y, Zhang Z, Tang L, Xu YC, Xie ZM, Gu XF, Wang HX (2012). Cytokine-induced killer cells in the treatment of patients with solid carcinomas: a systematic review and pooled analysis. Cytotherapy.

[10] Mesiano G, Todorovic M, Gammaitoni L, Leuci V, Giraudo Diego L, Carnevale-Schianca F, Fagioli F, Piacibello W, Aglietta M, Sangiolo D (2012). Cytokine-induced killer (CIK) cells as feasible and effective adoptive immunotherapy for the treatment of solid tumors. Expert Opin Biol Ther.

[11] Zhang Y, Xia L, Zhang Y, Wang Y, Lu X, Shi F, Liu Y, Chen M, Feng K, Zhang W, Fu X, Han W (2014). Analysis of adverse events following the treatment of autologous cytokine-induced killer cells for adoptive immunotherapy in malignant tumour sufferers. Expert Opin Biol Ther.

[12] Schmidt-Wolf IG, Finke S, Trojaneck B, Denkena A, Lefterova P, Schwella N, Heuft HG, Prange G, Korte M, Takeya M, Dorbic T, Neubauer A, Wittig B, Huhn D (1999). Phase I clinical study applying autologous immunological effector cells transfected with the interleukin-2 gene in patients with metastatic renal cancer, colorectal cancer and lymphoma. Br J Cancer.

[13] Wang ZX, Li JL, Cao JX, Liu YS, Li D, Zhang XY, Wang M, Wu M, Xu BL, Liu JL, Wang HB (2014). Cytokine-induced killer cells in the treatment of patients with renal cell carcinoma: a pooled meta-analysis. Immunotherapy.

[14] Wang H, Cao F, Li J, Li Y, Liu X, Wang L, Liu Z, Li Y, Zhao H, Zhou J (2014). Homing of cytokine-induced killer cells during the treatment of acute promyelocytic leukemia. Int J Hematol.

[15] Lee JH, Lee JH, Lim YS, Yeon JE, Song TJ, Yu SJ, Gwak GY, Kim KM, Kim YJ, Lee JW, Yoon JH (2015). Adjuvant immunotherapy with autologous cytokine-induced killer cells for hepatocellular carcinoma. Gastroenterology.

[16] Jin J, Joo KM, Lee SJ, Jo MY, Kim Y, Jin Y, Kim JK, Ahn JM, Yoon MJ, Lim J, Nam DH (2011). Synergistic therapeutic effects of cytokine-induced killer cells and temozolomide against glioblastoma. Oncol Rep.

[17] Takayama T, Sekine T, Makuuchi M, Yamasaki S, Kosuge T, Yamamoto J, Shimada K, Sakamoto M, Hirohashi S, Ohashi Y, Kakizoe T (2000). Adoptive immunotherapy to lower postsurgical recurrence rates of hepatocellular carcinoma: a randomised trial. Lancet.

[18] Macdonald DR, Cascino TL, Schold SC, Cairncross JG (1990). Response criteria for phase II studies of supratentorial malignant glioma. J Clin Oncol.

[19] Wen PY, Macdonald DR, Reardon DA, Cloughesy TF, Sorensen AG, Galanis E, Degroot J, Wick W, Gilbert MR, Lassman AB, Tsien C, Mikkelsen T, Wong ET (2010). Updated response assessment criteria for high-grade gliomas: response assessment in neuro-oncology working group. J Clin Oncol.

[20] Chinot OL, Wick W, Mason W, Henriksson R, Saran F, Nishikawa R, Carpentier AF, Hoang-Xuan K, Kavan P, Cernea D, Brandes AA, Hilton M, Abrey L, Cloughesy T (2014). Bevacizumab plus radiotherapy-temozolomide for newly diagnosed glioblastoma. N Engl J Med.

[21] Gilbert MR, Dignam JJ, Armstrong TS, Wefel JS, Blumenthal DT, Vogelbaum MA, Colman H, Chakravarti A, Pugh S, Won M, Jeraj R, Brown PD, Jaeckle KA (2014). A randomized trial of bevacizumab for newly diagnosed glioblastoma. N Engl J Med.

[22] Stupp R, Hegi ME, Gorlia T, Erridge SC, Perry J, Hong YK, Aldape KD, Lhermitte B, Pietsch T, Grujicic D, Steinbach JP, Wick W, Tarnawski R (2014). Cilengitide combined with standard treatment for patients with newly diagnosed glioblastoma with methylated MGMT promoter (CENTRIC EORTC 26071-22072 study): a multicentre, randomised, open-label, phase 3 trial. Lancet Oncol.

