# Supplementary material for: Phase III randomized trial of autologous cytokine-induced killer cell immunotherapy for newly diagnosed glioblastoma in korea
Source: Oncotarget. 2016 Sep 27;8(4):7003–13. doi: 10.18632/oncotarget.12273 (PMC5351686; doi:10.18632/oncotarget.12273)
Supplement: Supplementary file 1 [file oncotarget-08-7003-s001.pdf]

Table S1. Progression free survival (PFS)(PP set=140 patients)

	CIK immunotherapy group (N=69)	Control group (N=71)
Incidence rate (Death or PD), n(%)	60(86.957%)	61(85.915%)
PD, n(%)	59(85.507%)	60(84.507%)
Death, n(%)	1(1.449%)	1(1.408%)
Progression-Free survival rate (%)		
12 months(%) [95% CI]	28.99 [18.85, 39.89]	19.72 [11.44, 29.64]
18 months(%) [95% CI]	26.09 [16.43, 36.79]	18.08[11.44, 29.64]
24 months(%) [95% CI]	18.31 [10.15, 28.37]	12.65 [5.95, 22.00]
Progression-Free survival, Mean±SE	13.252±1.533	7.700±0.760
75% PFS [95% CI]	19.243 [9.737, 26.447]	8.586 [8.026, 19.474]
50% PFS [95% CI]	8.092 [7.072, 8.882]	5.362 [3.980, 7.928]
25% PFS [95% CI]	5.263 [2.697, 5.822]	2.467 [2.336, 2.763]
p-value[1)]	0.0218#	
Hazard ratio [90% CI][2)]	0.693 [0.512, 0.937]	

* Progression-free survival=Diagnosis date of disease progression by MRI, date of death or date of off-study - Randomization date

[1)] Results by one-sided Log-rank test

[2)] Presented as the result of one-sided test

P-value<0.05

Table S2. Overall survival (OS)(PP set=140 patients)

	CIK immunotherapy group (N=69)	Control group (N=71)
Incidence rate(Death), n(%)	42(60.870%)	43(60.563%)
Overall Survival rate(%)		
12 months(%) [95% CI]	80.54 [68.86, 88.21]	76.34 [64.30, 84.79]
18 months(%) [95% CI]	59.41[46.29, 70.33]	43.62[31.11, 55.46]
24 months(%) [95% CI]	40.92 [28.26, 53.16]	39.56 [27.21, 51.64]
Survival, Mean±SE	22.669±1.329	19.945±1.198
75% Survival [95% CI]	- [28.586, -]	- [25.428, -]
50% Survival [95% CI]	23.059 [17.204, 28.586]	16.875 [13.915, 24.211]
25% Survival [95% CI]	14.276 [9.013, 17.204]	12.007 [10.526, 13.651]
p-value[1)]	0.4221	
Hazard ratio [90% CI][2)]	0.840 [0.548, 1.287]	

* Overall Survival=Date of death or date of off-study - Randomization date

[1)] Results by Log-rank test

[2)] Presented as the result of one-sided test

Table S3. Response Rate(Objective Response rate, Disease Control Rate)

Treatment response rate

Treatment response	CIK group n	(%)	95% C.I. [min,	max]	Control group n	(%)	95% C.I. [min,	max]	*p*-value
ITT set	N=	91			N=	89			
n	85				82				
ORR	23	(27.059)	[17.610,	36.500]	13	(15.854)	[7.950,	23.760]	0.0783†
DCR	70	(82.353)	[74.250,	90.460]	52	(63.415)	[52.990,	73.840]	**0.0058†**
Complete response(CR)	7	(8.235)			4	(4.878)			
Partial response(PR)	16	(18.824)			9	(10.976)			
No change(NC)	47	(55.294)			39	(47.561)			
Progressive disease(PD)	15	(17.647)			30	(36.585)			

ORR=CR+PR

DCR=CR+PR+SD

†: Pearson's chi-square test

Arthralgia	6(7.059)	[6]	0(0.000)	[0]	0(0.000)	[0]	0(0.000)	[0]	8(9.412)	[8]	0(0.000)	[0]	0(0.000)	[0]	0(0.000)	[0]
Back pain	9(10.588)	[9]	2(2.353)	[2]	1(1.177)	[1]	1(1.177)	[1]	5(5.882)	[5]	0(0.000)	[0]	0(0.000)	[0]	0(0.000)	[0]
Myalgia	5(5.882)	[5]	0(0.000)	[0]	0(0.000)	[0]	0(0.000)	[0]	2(2.353)	[2]	0(0.000)	[0]	0(0.000)	[0]	0(0.000)	[0]
Muscular weakness	3(3.529)	[3]	0(0.000)	[0]	0(0.000)	[0]	0(0.000)	[0]	2(2.353)	[2]	0(0.000)	[0]	0(0.000)	[0]	0(0.000)	[0]
Neck pain	4(4.706)	[4]	0(0.000)	[0]	0(0.000)	[0]	0(0.000)	[0]	2(2.353)	[2]	0(0.000)	[0]	0(0.000)	[0]	0(0.000)	[0]
Flank pain	3(3.529)	[3]	0(0.000)	[0]	0(0.000)	[0]	0(0.000)	[0]	1(1.177)	[1]	0(0.000)	[0]	0(0.000)	[0]	0(0.000)	[0]

Musculoskeletal stiffness	2(2.353)	[2]	0(0.000)	[0]	0(0.000)	[0]	0(0.000)	[0]	2(2.353)	[2]	0(0.000)	[0]	0(0.000)	[0]	0(0.000)	[0]
Joint swelling	1(1.177)	[1]	0(0.000)	[0]	0(0.000)	[0]	0(0.000)	[0]	1(1.177)	[1]	0(0.000)	[0]	0(0.000)	[0]	0(0.000)	[0]
Muscle spasms	0(0.000)	[0]	0(0.000)	[0]	0(0.000)	[0]	0(0.000)	[0]	2(2.353)	[2]	0(0.000)	[0]	0(0.000)	[0]	0(0.000)	[0]
Periarthritis	0(0.000)	[0]	0(0.000)	[0]	0(0.000)	[0]	0(0.000)	[0]	2(2.353)	[2]	0(0.000)	[0]	0(0.000)	[0]	0(0.000)	[0]
Hypercreatinaemia	0(0.000)	[0]	0(0.000)	[0]	0(0.000)	[0]	0(0.000)	[0]	1(1.177)	[1]	0(0.000)	[0]	0(0.000)	[0]	0(0.000)	[0]
Musculoskeletal chest pain	1(1.177)	[1]	0(0.000)	[0]	0(0.000)	[0]	0(0.000)	[0]	0(0.000)	[0]	0(0.000)	[0]	0(0.000)	[0]	0(0.000)	[0]
Musculoskeletal discomfort	1(1.177)	[1]	0(0.000)	[0]	0(0.000)	[0]	0(0.000)	[0]	0(0.000)	[0]	0(0.000)	[0]	0(0.000)	[0]	0(0.000)	[0]
Neck mass	0(0.000)	[0]	0(0.000)	[0]	0(0.000)	[0]	0(0.000)	[0]	1(1.177)	[1]	0(0.000)	[0]	0(0.000)	[0]	0(0.000)	[0]
Osteoarthritis	0(0.000)	[0]	0(0.000)	[0]	0(0.000)	[0]	0(0.000)	[0]	1(1.177)	[1]	0(0.000)	[0]	0(0.000)	[0]	0(0.000)	[0]
Osteopenia	0(0.000)	[0]	0(0.000)	[0]	0(0.000)	[0]	0(0.000)	[0]	1(1.177)	[1]	0(0.000)	[0]	0(0.000)	[0]	0(0.000)	[0]
Osteoporosis	1(1.177)	[1]	0(0.000)	[0]	0(0.000)	[0]	0(0.000)	[0]	0(0.000)	[0]	0(0.000)	[0]	0(0.000)	[0]	0(0.000)	[0]
Sensation of heaviness	1(1.177)	[1]	0(0.000)	[0]	0(0.000)	[0]	0(0.000)	[0]	0(0.000)	[0]	0(0.000)	[0]	0(0.000)	[0]	0(0.000)	[0]
**Investigations**	**36(42.353)**	**[109]**	**11(12.941)**	**[23]**	**11(12.941)**	**[17]**	**3(3.529)**	**[6]**	**24(28.235)**	**[40]**	**5(5.882)**	**[5]**	**4(4.706)**	**[4]**	**1(1.176)**	**[1]**
Alanine aminotransferase increased	15(17.647)	[19]	2(2.353)	[2]	2(2.353)	[2]	0(0.000)	[0]	10(11.765)	[11]	0(0.000)	[0]	0(0.000)	[0]	0(0.000)	[0]
Neutrophil count decreased	15(17.647)	[19]	7(8.235)	[9]	6(7.059)	[8]	1(1.177)	[1]	7(8.235)	[8]	3(3.529)	[3]	2(2.353)	[2]	1(1.176)	[1]
Aspartate aminotransferase increased	8(9.412)	[9]	1(1.177)	[1]	0(0.000)	[0]	1(1.177)	[1]	6(7.059)	[7]	1(1.177)	[1]	1(1.177)	[1]	0(0.000)	[0]
Platelet count decreased	9(10.588)	[12]	3(3.529)	[4]	2(2.353)	[2]	1(1.177)	[2]	3(3.529)	[3]	0(0.000)	[0]	0(0.000)	[0]	0(0.000)	[0]
White blood cell count decreased	8(9.412)	[18]	3(3.529)	[5]	3(3.529)	[4]	1(1.177)	[1]	2(2.353)	[3]	0(0.000)	[0]	0(0.000)	[0]	0(0.000)	[0]
Haemoglobin decreased	5(5.882)	[5]	1(1.177)	[1]	0(0.000)	[0]	1(1.177)	[1]	2(2.353)	[2]	0(0.000)	[0]	0(0.000)	[0]	0(0.000)	[0]
Hepatic enzyme increased	2(2.353)	[2]	0(0.000)	[0]	0(0.000)	[0]	0(0.000)	[0]	2(2.353)	[2]	0(0.000)	[0]	0(0.000)	[0]	0(0.000)	[0]
Blood sodium decreased	1(1.177)	[1]	0(0.000)	[0]	0(0.000)	[0]	0(0.000)	[0]	2(2.353)	[2]	1(1.177)	[1]	1(1.177)	[1]	0(0.000)	[0]
Weight decreased	2(2.353)	[2]	0(0.000)	[0]	0(0.000)	[0]	0(0.000)	[0]	1(1.177)	[1]	0(0.000)	[0]	0(0.000)	[0]	0(0.000)	[0]
Ammonia increased	1(1.177)	[1]	0(0.000)	[0]	0(0.000)	[0]	0(0.000)	[0]	1(1.177)	[1]	0(0.000)	[0]	0(0.000)	[0]	0(0.000)	[0]
Skin test positive	2(2.353)	[15]	0(0.000)	[0]	0(0.000)	[0]	0(0.000)	[0]	0(0.000)	[0]	0(0.000)	[0]	0(0.000)	[0]	0(0.000)	[0]
Blood lactate dehydrogenase increased	1(1.177)	[1]	0(0.000)	[0]	0(0.000)	[0]	0(0.000)	[0]	0(0.000)	[0]	0(0.000)	[0]	0(0.000)	[0]	0(0.000)	[0]
Blood potassium decreased	1(1.177)	[1]	0(0.000)	[0]	0(0.000)	[0]	0(0.000)	[0]	0(0.000)	[0]	0(0.000)	[0]	0(0.000)	[0]	0(0.000)	[0]

Clostridium test positive	1(1.177)	[1]	0(0.000)	[0]	0(0.000)	[0]	0(0.000)	[0]	0(0.000)	[0]	0(0.000)	[0]	0(0.000)	[0]	0(0.000)	[0]
Electrocardiogram QT prolonged	1(1.177)	[1]	0(0.000)	[0]	0(0.000)	[0]	0(0.000)	[0]	0(0.000)	[0]	0(0.000)	[0]	0(0.000)	[0]	0(0.000)	[0]
Electrocardiogram T wave abnormal	1(1.177)	[1]	0(0.000)	[0]	0(0.000)	[0]	0(0.000)	[0]	0(0.000)	[0]	0(0.000)	[0]	0(0.000)	[0]	0(0.000)	[0]
Lymphocyte count decreased	1(1.177)	[1]	1(1.177)	[1]	1(1.177)	[1]	0(0.000)	[0]	0(0.000)	[0]	0(0.000)	[0]	0(0.000)	[0]	0(0.000)	[0]
**Respiratory, thoracic and mediastinal disorders**	**28(32.941)**	**[46]**	**3(3.529)**	**[4]**	**3(3.529)**	**[4]**	**0(0.000)**	**[0]**	**21(24.706)**	**[33]**	**2(2.353)**	**[2]**	**1(1.177)**	**[1]**	**1(1.176)**	**[1]**
Cough	9(10.588)	[9]	0(0.000)	[0]	0(0.000)	[0]	0(0.000)	[0]	7(8.235)	[8]	0(0.000)	[0]	0(0.000)	[0]	0(0.000)	[0]
Dyspnoea	9(10.588)	[11]	2(2.353)	[2]	2(2.353)	[2]	0(0.000)	[0]	5(5.882)	[5]	1(1.177)	[1]	0(0.000)	[0]	1(1.176)	[1]
Productive cough	9(10.588)	[9]	0(0.000)	[0]	0(0.000)	[0]	0(0.000)	[0]	4(4.706)	[5]	0(0.000)	[0]	0(0.000)	[0]	0(0.000)	[0]
Oropharyngeal pain	5(5.882)	[5]	0(0.000)	[0]	0(0.000)	[0]	0(0.000)	[0]	1(1.177)	[1]	0(0.000)	[0]	0(0.000)	[0]	0(0.000)	[0]
Rhinorrhoea	2(2.353)	[2]	0(0.000)	[0]	0(0.000)	[0]	0(0.000)	[0]	3(3.529)	[3]	0(0.000)	[0]	0(0.000)	[0]	0(0.000)	[0]
Hiccups	1(1.177)	[1]	0(0.000)	[0]	0(0.000)	[0]	0(0.000)	[0]	2(2.353)	[5]	1(1.177)	[1]	1(1.177)	[1]	0(0.000)	[0]
Epistaxis	1(1.177)	[1]	0(0.000)	[0]	0(0.000)	[0]	0(0.000)	[0]	1(1.177)	[1]	0(0.000)	[0]	0(0.000)	[0]	0(0.000)	[0]
Aspiration	1(1.177)	[1]	0(0.000)	[0]	0(0.000)	[0]	0(0.000)	[0]	0(0.000)	[0]	0(0.000)	[0]	0(0.000)	[0]	0(0.000)	[0]
Atelectasis	1(1.177)	[1]	1(1.177)	[1]	1(1.177)	[1]	0(0.000)	[0]	0(0.000)	[0]	0(0.000)	[0]	0(0.000)	[0]	0(0.000)	[0]
Bronchitis chronic	1(1.177)	[1]	0(0.000)	[0]	0(0.000)	[0]	0(0.000)	[0]	0(0.000)	[0]	0(0.000)	[0]	0(0.000)	[0]	0(0.000)	[0]
Dyspnoea exertional	1(1.177)	[1]	0(0.000)	[0]	0(0.000)	[0]	0(0.000)	[0]	0(0.000)	[0]	0(0.000)	[0]	0(0.000)	[0]	0(0.000)	[0]
Emphysema	0(0.000)	[0]	0(0.000)	[0]	0(0.000)	[0]	0(0.000)	[0]	1(1.177)	[1]	0(0.000)	[0]	0(0.000)	[0]	0(0.000)	[0]
Haemoptysis	0(0.000)	[0]	0(0.000)	[0]	0(0.000)	[0]	0(0.000)	[0]	1(1.177)	[1]	0(0.000)	[0]	0(0.000)	[0]	0(0.000)	[0]
Increased bronchial secretion	0(0.000)	[0]	0(0.000)	[0]	0(0.000)	[0]	0(0.000)	[0]	1(1.177)	[1]	0(0.000)	[0]	0(0.000)	[0]	0(0.000)	[0]
Pneumonia aspiration	1(1.177)	[1]	1(1.177)	[1]	1(1.177)	[1]	0(0.000)	[0]	0(0.000)	[0]	0(0.000)	[0]	0(0.000)	[0]	0(0.000)	[0]
Pneumothorax	0(0.000)	[0]	0(0.000)	[0]	0(0.000)	[0]	0(0.000)	[0]	1(1.177)	[1]	0(0.000)	[0]	0(0.000)	[0]	0(0.000)	[0]
Pulmonary hypertension	1(1.177)	[1]	0(0.000)	[0]	0(0.000)	[0]	0(0.000)	[0]	0(0.000)	[0]	0(0.000)	[0]	0(0.000)	[0]	0(0.000)	[0]
Pulmonary oedema	1(1.177)	[1]	0(0.000)	[0]	0(0.000)	[0]	0(0.000)	[0]	0(0.000)	[0]	0(0.000)	[0]	0(0.000)	[0]	0(0.000)	[0]
Rhinitis allergic	0(0.000)	[0]	0(0.000)	[0]	0(0.000)	[0]	0(0.000)	[0]	1(1.177)	[1]	0(0.000)	[0]	0(0.000)	[0]	0(0.000)	[0]
Sleep apnoea syndrome	1(1.177)	[1]	0(0.000)	[0]	0(0.000)	[0]	0(0.000)	[0]	0(0.000)	[0]	0(0.000)	[0]	0(0.000)	[0]	0(0.000)	[0]
**Blood and lymphatic system disorders**	**24(28.235)**	**[58]**	**10(11.765)**	**[18]**	**10(11.765)**	**[16]**	**1(1.177)**	**[2]**	**16(18.824)**	**[30]**	**4(4.706)**	**[5]**	**4(4.706)**	**[5]**	**0(0.000)**	**[0]**
Thrombocytopenia	13(15.294)	[15]	2(2.353)	[2]	1(1.177)	[1]	1(1.177)	[1]	12(14.118)	[13]	1(1.177)	[1]	1(1.177)	[1]	0(0.000)	[0]

Leukopenia	16(18.824)	[26]	2(2.353)	[2]	1(1.177)	[1]	1(1.177)	[1]	7(8.235)	[8]	2(2.353)	[2]	2(2.353)	[2]	0(0.000)	[0]
Neutropenia	4(4.706)	[8]	3(3.529)	[7]	3(3.529)	[7]	0(0.000)	[0]	3(3.529)	[3]	0(0.000)	[0]	0(0.000)	[0]	0(0.000)	[0]
Lymphopenia	4(4.706)	[6]	4(4.706)	[5]	4(4.706)	[5]	0(0.000)	[0]	2(2.353)	[3]	1(1.177)	[2]	1(1.177)	[2]	0(0.000)	[0]
Anaemia	1(1.177)	[1]	0(0.000)	[0]	0(0.000)	[0]	0(0.000)	[0]	2(2.353)	[2]	0(0.000)	[0]	0(0.000)	[0]	0(0.000)	[0]
Disseminated intravascular coagulation	2(2.353)	[2]	2(2.353)	[2]	2(2.353)	[2]	0(0.000)	[0]	0(0.000)	[0]	0(0.000)	[0]	0(0.000)	[0]	0(0.000)	[0]
Splenomegaly	0(0.000)	[0]	0(0.000)	[0]	0(0.000)	[0]	0(0.000)	[0]	1(1.177)	[1]	0(0.000)	[0]	0(0.000)	[0]	0(0.000)	[0]
**Eye disorders**	**19(22.353)**	**[21]**	**1(1.177)**	**[1]**	**1(1.177)**	**[1]**	**0(0.000)**	**[0]**	**21(24.706)**	**[26]**	**0(0.000)**	**[0]**	**0(0.000)**	**[0]**	**0(0.000)**	**[0]**
Dry eye	6(7.059)	[6]	0(0.000)	[0]	0(0.000)	[0]	0(0.000)	[0]	2(2.353)	[2]	0(0.000)	[0]	0(0.000)	[0]	0(0.000)	[0]
Eye pain	1(1.177)	[1]	0(0.000)	[0]	0(0.000)	[0]	0(0.000)	[0]	6(7.059)	[6]	0(0.000)	[0]	0(0.000)	[0]	0(0.000)	[0]
Diplopia	1(1.177)	[1]	0(0.000)	[0]	0(0.000)	[0]	0(0.000)	[0]	3(3.529)	[3]	0(0.000)	[0]	0(0.000)	[0]	0(0.000)	[0]
Eyelid oedema	0(0.000)	[0]	0(0.000)	[0]	0(0.000)	[0]	0(0.000)	[0]	4(4.706)	[4]	0(0.000)	[0]	0(0.000)	[0]	0(0.000)	[0]
Visual impairment	2(2.353)	[2]	0(0.000)	[0]	0(0.000)	[0]	0(0.000)	[0]	3 (3.529)	[3]	0(0.000)	[0]	0(0.000)	[0]	0(0.000)	[0]
Conjunctivitis	1(1.177)	[1]	0(0.000)	[0]	0(0.000)	[0]	0(0.000)	[0]	2(2.353)	[2]	0(0.000)	[0]	0(0.000)	[0]	0(0.000)	[0]
Foreign body sensation in eyes	1(1.177)	[1]	0(0.000)	[0]	0(0.000)	[0]	0(0.000)	[0]	1(1.177)	[1]	0(0.000)	[0]	0(0.000)	[0]	0(0.000)	[0]
Ocular hyperaemia	2(2.353)	[2]	0(0.000)	[0]	0(0.000)	[0]	0(0.000)	[0]	0(0.000)	[0]	0(0.000)	[0]	0(0.000)	[0]	0(0.000)	[0]
Vision blurred	1(1.177)	[1]	0(0.000)	[0]	0(0.000)	[0]	0(0.000)	[0]	1(1.177)	[1]	0(0.000)	[0]	0(0.000)	[0]	0(0.000)	[0]
Cataract	1(1.177)	[1]	1(1.177)	[1]	1(1.177)	[1]	0(0.000)	[0]	0(0.000)	[0]	0(0.000)	[0]	0(0.000)	[0]	0(0.000)	[0]
Corneal erosion	0(0.000)	[0]	0(0.000)	[0]	0(0.000)	[0]	0(0.000)	[0]	1(1.177)	[1]	0(0.000)	[0]	0(0.000)	[0]	0(0.000)	[0]
Eye inflammation	1(1.177)	[1]	0(0.000)	[0]	0(0.000)	[0]	0(0.000)	[0]	0(0.000)	[0]	0(0.000)	[0]	0(0.000)	[0]	0(0.000)	[0]
Eye oedema	1(1.177)	[1]	0(0.000)	[0]	0(0.000)	[0]	0(0.000)	[0]	0(0.000)	[0]	0(0.000)	[0]	0(0.000)	[0]	0(0.000)	[0]
Eye swelling	0(0.000)	[0]	0(0.000)	[0]	0(0.000)	[0]	0(0.000)	[0]	1(1.177)	[1]	0(0.000)	[0]	0(0.000)	[0]	0(0.000)	[0]
Glare	1(1.177)	[1]	0(0.000)	[0]	0(0.000)	[0]	0(0.000)	[0]	0(0.000)	[0]	0(0.000)	[0]	0(0.000)	[0]	0(0.000)	[0]
Lacrimation increased	1(1.177)	[1]	0(0.000)	[0]	0(0.000)	[0]	0(0.000)	[0]	0(0.000)	[0]	0(0.000)	[0]	0(0.000)	[0]	0(0.000)	[0]
Ocular discomfort	0(0.000)	[0]	0(0.000)	[0]	0(0.000)	[0]	0(0.000)	[0]	1(1.177)	[1]	0(0.000)	[0]	0(0.000)	[0]	0(0.000)	[0]
Retinal degeneration	1(1.177)	[1]	0(0.000)	[0]	0(0.000)	[0]	0(0.000)	[0]	0(0.000)	[0]	0(0.000)	[0]	0(0.000)	[0]	0(0.000)	[0]
Visual acuity reduced	0(0.000)	[0]	0(0.000)	[0]	0(0.000)	[0]	0(0.000)	[0]	1(1.177)	[1]	0(0.000)	[0]	0(0.000)	[0]	0(0.000)	[0]
**Renal and urinary disorders**	**18(21.177)**	**[26]**	**3(3.529)**	**[3]**	**3(3.529)**	**[3]**	**0(0.000)**	**[0]**	**12(14.118)**	**[13]**	**1(1.177)**	**[1]**	**1(1.177)**	**[1]**	**0(0.000)**	**[0]**
Urinary incontinence	3(3.529)	[3]	0(0.000)	[0]	0(0.000)	[0]	0(0.000)	[0]	5(5.882)	[5]	0(0.000)	[0]	0(0.000)	[0]	0(0.000)	[0]

Dysuria	6(7.059)	[6]	1(1.177)	[1]	1(1.177)	[1]	0(0.000)	[0]	1(1.177)	[1]	0(0.000)	[0]	0(0.000)	[0]	0(0.000)	[0]
Haematuria	5(5.882)	[5]	0(0.000)	[0]	0(0.000)	[0]	0(0.000)	[0]	1(1.177)	[1]	0(0.000)	[0]	0(0.000)	[0]	0(0.000)	[0]
Micturition disorder	2(2.353)	[2]	0(0.000)	[0]	0(0.000)	[0]	0(0.000)	[0]	1(1.177)	[1]	0(0.000)	[0]	0(0.000)	[0]	0(0.000)	[0]
Pollakiuria	2(2.353)	[2]	0(0.000)	[0]	0(0.000)	[0]	0(0.000)	[0]	1(1.177)	[1]	0(0.000)	[0]	0(0.000)	[0]	0(0.000)	[0]
Neurogenic bladder	0(0.000)	[0]	0(0.000)	[0]	0(0.000)	[0]	0(0.000)	[0]	2(2.353)	[2]	1(1.177)	[1]	1(1.177)	[1]	0(0.000)	[0]
Urinary retention	2(2.353)	[2]	0(0.000)	[0]	0(0.000)	[0]	0(0.000)	[0]	0(0.000)	[0]	0(0.000)	[0]	0(0.000)	[0]	0(0.000)	[0]
Calculus bladder	1(1.177)	[1]	0(0.000)	[0]	0(0.000)	[0]	0(0.000)	[0]	0(0.000)	[0]	0(0.000)	[0]	0(0.000)	[0]	0(0.000)	[0]
Enuresis	0(0.000)	[0]	0(0.000)	[0]	0(0.000)	[0]	0(0.000)	[0]	1(1.177)	[1]	0(0.000)	[0]	0(0.000)	[0]	0(0.000)	[0]
Glycosuria	1(1.177)	[1]	0(0.000)	[0]	0(0.000)	[0]	0(0.000)	[0]	0(0.000)	[0]	0(0.000)	[0]	0(0.000)	[0]	0(0.000)	[0]
Hypertonic bladder	1(1.177)	[1]	0(0.000)	[0]	0(0.000)	[0]	0(0.000)	[0]	0(0.000)	[0]	0(0.000)	[0]	0(0.000)	[0]	0(0.000)	[0]
Micturition urgency	0(0.000)	[0]	0(0.000)	[0]	0(0.000)	[0]	0(0.000)	[0]	1(1.177)	[1]	0(0.000)	[0]	0(0.000)	[0]	0(0.000)	[0]
Oliguria	1(1.177)	[1]	0(0.000)	[0]	0(0.000)	[0]	0(0.000)	[0]	0(0.000)	[0]	0(0.000)	[0]	0(0.000)	[0]	0(0.000)	[0]
Renal failure acute	1(1.177)	[1]	1(1.177)	[1]	1(1.177)	[1]	0(0.000)	[0]	0(0.000)	[0]	0(0.000)	[0]	0(0.000)	[0]	0(0.000)	[0]
Renal tubular necrosis	1(1.177)	[1]	1(1.177)	[1]	1(1.177)	[1]	0(0.000)	[0]	0(0.000)	[0]	0(0.000)	[0]	0(0.000)	[0]	0(0.000)	[0]
**Psychiatric disorders**	**14(16.471)**	**[17]**	**0(0.000)**	**[0]**	**0(0.000)**	**[0]**	**0(0.000)**	**[0]**	**16 (18.824)**	**[22]**	**0(0.000)**	**[0]**	**0(0.000)**	**[0]**	**0(0.000)**	**[0]**
Insomnia	7(8.235)	[9]	0(0.000)	[0]	0(0.000)	[0]	0(0.000)	[0]	7(8.235)	[7]	0(0.000)	[0]	0(0.000)	[0]	0(0.000)	[0]
Depression	4(4.706)	[4]	0(0.000)	[0]	0(0.000)	[0]	0(0.000)	[0]	6 (7.059)	[7]	0(0.000)	[0]	0(0.000)	[0]	0(0.000)	[0]
Mood altered	1(1.177)	[1]	0(0.000)	[0]	0(0.000)	[0]	0(0.000)	[0]	2(2.353)	[2]	0(0.000)	[0]	0(0.000)	[0]	0(0.000)	[0]
Psychotic disorder	0(0.000)	[0]	0(0.000)	[0]	0(0.000)	[0]	0(0.000)	[0]	2(2.353)	[2]	0(0.000)	[0]	0(0.000)	[0]	0(0.000)	[0]
Anxiety disorder	0(0.000)	[0]	0(0.000)	[0]	0(0.000)	[0]	0(0.000)	[0]	1(1.177)	[1]	0(0.000)	[0]	0(0.000)	[0]	0(0.000)	[0]
Depressed mood	1(1.177)	[1]	0(0.000)	[0]	0(0.000)	[0]	0(0.000)	[0]	1 (1.177)	[1]	0(0.000)	[0]	0(0.000)	[0]	0(0.000)	[0]
Personality change	0(0.000)	[0]	0(0.000)	[0]	0(0.000)	[0]	0(0.000)	[0]	1(1.177)	[1]	0(0.000)	[0]	0(0.000)	[0]	0(0.000)	[0]
Personality disorder	0(0.000)	[0]	0(0.000)	[0]	0(0.000)	[0]	0(0.000)	[0]	1(1.177)	[1]	0(0.000)	[0]	0(0.000)	[0]	0(0.000)	[0]
Sleep disorder	1(1.177)	[2]	0(0.000)	[0]	0(0.000)	[0]	0(0.000)	[0]	0(0.000)	[0]	0(0.000)	[0]	0(0.000)	[0]	0(0.000)	[0]
**Injury, poisoning and procedural complications**	**16(18.824)**	**[22]**	**0(0.000)**	**[0]**	**0(0.000)**	**[0]**	**0(0.000)**	**[0]**	**7(8.235)**	**[8]**	**0(0.000)**	**[0]**	**0(0.000)**	**[0]**	**0(0.000)**	**[0]**
Excoriation	4(4.706)	[4]	0(0.000)	[0]	0(0.000)	[0]	0(0.000)	[0]	2(2.353)	[2]	0(0.000)	[0]	0(0.000)	[0]	0(0.000)	[0]
Ear abrasion	3(3.529)	[3]	0(0.000)	[0]	0(0.000)	[0]	0(0.000)	[0]	1(1.177)	[1]	0(0.000)	[0]	0(0.000)	[0]	0(0.000)	[0]
Procedural pain	1(1.177)	[1]	0(0.000)	[0]	0(0.000)	[0]	0(0.000)	[0]	2(2.353)	[2]	0(0.000)	[0]	0(0.000)	[0]	0(0.000)	[0]

Contusion	1(1.177)	[3]	0(0.000)	[0]	0(0.000)	[0]	0(0.000)	[0]	1(1.177)	[1]	0(0.000)	[0]	0(0.000)	[0]	0(0.000)	[0]
Laceration	2(2.353)	[2]	0(0.000)	[0]	0(0.000)	[0]	0(0.000)	[0]	0(0.000)	[0]	0(0.000)	[0]	0(0.000)	[0]	0(0.000)	[0]
Rib fracture	2(2.353)	[2]	0(0.000)	[0]	0(0.000)	[0]	0(0.000)	[0]	0(0.000)	[0]	0(0.000)	[0]	0(0.000)	[0]	0(0.000)	[0]
Concussion	1(1.177)	[1]	0(0.000)	[0]	0(0.000)	[0]	0(0.000)	[0]	0(0.000)	[0]	0(0.000)	[0]	0(0.000)	[0]	0(0.000)	[0]
Femur fracture	1(1.177)	[1]	0(0.000)	[0]	0(0.000)	[0]	0(0.000)	[0]	0(0.000)	[0]	0(0.000)	[0]	0(0.000)	[0]	0(0.000)	[0]
Hand fracture	1(1.177)	[1]	0(0.000)	[0]	0(0.000)	[0]	0(0.000)	[0]	0(0.000)	[0]	0(0.000)	[0]	0(0.000)	[0]	0(0.000)	[0]
Hip fracture	1(1.177)	[1]	0(0.000)	[0]	0(0.000)	[0]	0(0.000)	[0]	0(0.000)	[0]	0(0.000)	[0]	0(0.000)	[0]	0(0.000)	[0]
Joint dislocation	1(1.177)	[1]	0(0.000)	[0]	0(0.000)	[0]	0(0.000)	[0]	0(0.000)	[0]	0(0.000)	[0]	0(0.000)	[0]	0(0.000)	[0]
Post procedural oedema	1(1.177)	[1]	0(0.000)	[0]	0(0.000)	[0]	0(0.000)	[0]	0(0.000)	[0]	0(0.000)	[0]	0(0.000)	[0]	0(0.000)	[0]
Radiation skin injury	1(1.177)	[1]	0(0.000)	[0]	0(0.000)	[0]	0(0.000)	[0]	0(0.000)	[0]	0(0.000)	[0]	0(0.000)	[0]	0(0.000)	[0]
Skin injury	0(0.000)	[0]	0(0.000)	[0]	0(0.000)	[0]	0(0.000)	[0]	1(1.177)	[1]	0(0.000)	[0]	0(0.000)	[0]	0(0.000)	[0]
Subdural haemorrhage	0(0.000)	[0]	0(0.000)	[0]	0(0.000)	[0]	0(0.000)	[0]	1(1.177)	[1]	0(0.000)	[0]	0(0.000)	[0]	0(0.000)	[0]
**Vascular disorders**	**8(9.412)**	**[9]**	**0(0.000)**	**[0]**	**0(0.000)**	**[0]**	**0(0.000)**	**[0]**	**4(4.706)**	**[5]**	**0(0.000)**	**[0]**	**0(0.000)**	**[0]**	**0(0.000)**	**[0]**
Hypotension	4(4.706)	[5]	0(0.000)	[0]	0(0.000)	[0]	0(0.000)	[0]	1(1.177)	[1]	0(0.000)	[0]	0(0.000)	[0]	0(0.000)	[0]
Flushing	1(1.177)	[1]	0(0.000)	[0]	0(0.000)	[0]	0(0.000)	[0]	2(2.353)	[2]	0(0.000)	[0]	0(0.000)	[0]	0(0.000)	[0]
Hypertension	1(1.177)	[1]	0(0.000)	[0]	0(0.000)	[0]	0(0.000)	[0]	2(2.353)	[2]	0(0.000)	[0]	0(0.000)	[0]	0(0.000)	[0]
Shock	1(1.177)	[1]	0(0.000)	[0]	0(0.000)	[0]	0(0.000)	[0]	0(0.000)	[0]	0(0.000)	[0]	0(0.000)	[0]	0(0.000)	[0]
Thrombosis	1(1.177)	[1]	0(0.000)	[0]	0(0.000)	[0]	0(0.000)	[0]	0(0.000)	[0]	0(0.000)	[0]	0(0.000)	[0]	0(0.000)	[0]
**Ear and labyrinth disorders**	**7(8.235)**	**[12]**	**0(0.000)**	**[0]**	**0(0.000)**	**[0]**	**0(0.000)**	**[0]**	**4(4.706)**	**[6]**	**0(0.000)**	**[0]**	**0(0.000)**	**[0]**	**0(0.000)**	**[0]**
Tinnitus	3(3.529)	[3]	0(0.000)	[0]	0(0.000)	[0]	0(0.000)	[0]	2(2.353)	[3]	0(0.000)	[0]	0(0.000)	[0]	0(0.000)	[0]
Ear pain	2(2.353)	[2]	0(0.000)	[0]	0(0.000)	[0]	0(0.000)	[0]	1(1.177)	[1]	0(0.000)	[0]	0(0.000)	[0]	0(0.000)	[0]
Otorrhoea	2(2.353)	[4]	0(0.000)	[0]	0(0.000)	[0]	0(0.000)	[0]	1(1.177)	[1]	0(0.000)	[0]	0(0.000)	[0]	0(0.000)	[0]
Deafness	1(1.177)	[1]	0(0.000)	[0]	0(0.000)	[0]	0(0.000)	[0]	1(1.177)	[1]	0(0.000)	[0]	0(0.000)	[0]	0(0.000)	[0]
Auricular swelling	1(1.177)	[1]	0(0.000)	[0]	0(0.000)	[0]	0(0.000)	[0]	0(0.000)	[0]	0(0.000)	[0]	0(0.000)	[0]	0(0.000)	[0]
Ear discomfort	1(1.177)	[1]	0(0.000)	[0]	0(0.000)	[0]	0(0.000)	[0]	0(0.000)	[0]	0(0.000)	[0]	0(0.000)	[0]	0(0.000)	[0]
**Reproductive system and breast disorders**	**5(5.882)**	**[6]**	**0(0.000)**	**[0]**	**0(0.000)**	**[0]**	**0(0.000)**	**[0]**	**2(2.353)**	**[2]**	**0(0.000)**	**[0]**	**0(0.000)**	**[0]**	**0(0.000)**	**[0]**
Vaginal haemorrhage	2(2.353)	[2]	0(0.000)	[0]	0(0.000)	[0]	0(0.000)	[0]	0(0.000)	[0]	0(0.000)	[0]	0(0.000)	[0]	0(0.000)	[0]
Galactorrhoea	0(0.000)	[0]	0(0.000)	[0]	0(0.000)	[0]	0(0.000)	[0]	1(1.177)	[1]	0(0.000)	[0]	0(0.000)	[0]	0(0.000)	[0]

Genital discharge	1(1.177)	[1]	0(0.000)	[0]	0(0.000)	[0]	0(0.000)	[0]	0(0.000)	[0]	0(0.000)	[0]	0(0.000)	[0]	0(0.000)	[0]
Menopausal symptoms	1(1.177)	[1]	0(0.000)	[0]	0(0.000)	[0]	0(0.000)	[0]	0(0.000)	[0]	0(0.000)	[0]	0(0.000)	[0]	0(0.000)	[0]
Menorrhagia	1(1.177)	[1]	0(0.000)	[0]	0(0.000)	[0]	0(0.000)	[0]	0(0.000)	[0]	0(0.000)	[0]	0(0.000)	[0]	0(0.000)	[0]
Perineal pain	0(0.000)	[0]	0(0.000)	[0]	0(0.000)	[0]	0(0.000)	[0]	1(1.177)	[1]	0(0.000)	[0]	0(0.000)	[0]	0(0.000)	[0]
Vulvovaginal swelling	1(1.177)	[1]	0(0.000)	[0]	0(0.000)	[0]	0(0.000)	[0]	0(0.000)	[0]	0(0.000)	[0]	0(0.000)	[0]	0(0.000)	[0]
**Immune system disorders**	**1(1.177)**	**[4]**	**0(0.000)**	**[0]**	**0(0.000)**	**[0]**	**0(0.000)**	**[0]**	**4(4.706)**	**[4]**	**0(0.000)**	**[0]**	**0(0.000)**	**[0]**	**0(0.000)**	**[0]**
Hypersensitivity	1(1.177)	[4]	0(0.000)	[0]	0(0.000)	[0]	0(0.000)	[0]	4(4.706)	[4]	0(0.000)	[0]	0(0.000)	[0]	0(0.000)	[0]
**Hepatobiliary disorders**	**0(0.000)**	**[0]**	**0(0.000)**	**[0]**	**0(0.000)**	**[0]**	**0(0.000)**	**[0]**	**4(4.706)**	**[5]**	**1(1.177)**	**[1]**	**1(1.177)**	**[1]**	**0(0.000)**	**[0]**
Hepatic steatosis	0(0.000)	[0]	0(0.000)	[0]	0(0.000)	[0]	0(0.000)	[0]	2(2.353)	[2]	0(0.000)	[0]	0(0.000)	[0]	0(0.000)	[0]
Hepatic cirrhosis	0(0.000)	[0]	0(0.000)	[0]	0(0.000)	[0]	0(0.000)	[0]	1(1.177)	[1]	0(0.000)	[0]	0(0.000)	[0]	0(0.000)	[0]
Hepatitis	0(0.000)	[0]	0(0.000)	[0]	0(0.000)	[0]	0(0.000)	[0]	1(1.177)	[1]	0(0.000)	[0]	0(0.000)	[0]	0(0.000)	[0]
Hepatitis acute	0(0.000)	[0]	0(0.000)	[0]	0(0.000)	[0]	0(0.000)	[0]	1(1.177)	[1]	1(1.177)	[1]	1(1.177)	[1]	0(0.000)	[0]
**Cardiac disorders**	**3(3.529)**	**[3]**	**0(0.000)**	**[0]**	**0(0.000)**	**[0]**	**0(0.000)**	**[0]**	**1(1.177)**	**[1]**	**0(0.000)**	**[0]**	**0(0.000)**	**[0]**	**0(0.000)**	**[0]**
Arrhythmia	1(1.177)	[1]	0(0.000)	[0]	0(0.000)	[0]	0(0.000)	[0]	0(0.000)	[0]	0(0.000)	[0]	0(0.000)	[0]	0(0.000)	[0]
Bradycardia	0(0.000)	[0]	0(0.000)	[0]	0(0.000)	[0]	0(0.000)	[0]	1(1.177)	[1]	0(0.000)	[0]	0(0.000)	[0]	0(0.000)	[0]
Tachycardia	1(1.177)	[1]	0(0.000)	[0]	0(0.000)	[0]	0(0.000)	[0]	0(0.000)	[0]	0(0.000)	[0]	0(0.000)	[0]	0(0.000)	[0]
Ventricular dysfunction	1(1.177)	[1]	0(0.000)	[0]	0(0.000)	[0]	0(0.000)	[0]	0(0.000)	[0]	0(0.000)	[0]	0(0.000)	[0]	0(0.000)	[0]
**Endocrine disorders**	**0(0.000)**	**[0]**	**0(0.000)**	**[0]**	**0(0.000)**	**[0]**	**0(0.000)**	**[0]**	**3(3.529)**	**[3]**	**0(0.000)**	**[0]**	**0(0.000)**	**[0]**	**0(0.000)**	**[0]**
Cushing's syndrome	0(0.000)	[0]	0(0.000)	[0]	0(0.000)	[0]	0(0.000)	[0]	1(1.177)	[1]	0(0.000)	[0]	0(0.000)	[0]	0(0.000)	[0]
Cushingoid	0(0.000)	[0]	0(0.000)	[0]	0(0.000)	[0]	0(0.000)	[0]	1(1.177)	[1]	0(0.000)	[0]	0(0.000)	[0]	0(0.000)	[0]
Thyroid mass	0(0.000)	[0]	0(0.000)	[0]	0(0.000)	[0]	0(0.000)	[0]	1(1.177)	[1]	0(0.000)	[0]	0(0.000)	[0]	0(0.000)	[0]
**Neoplasms benign, malignant and unspecified (incl cysts and polyps)**	**0(0.000)**	**[0]**	**0(0.000)**	**[0]**	**0(0.000)**	**[0]**	**0(0.000)**	**[0]**	**1(1.177)**	**[1]**	**0(0.000)**	**[0]**	**0(0.000)**	**[0]**	**0(0.000)**	**[0]**
Uterine leiomyoma	0(0.000)	[0]	0(0.000)	[0]	0(0.000)	[0]	0(0.000)	[0]	1(1.177)	[1]	0(0.000)	[0]	0(0.000)	[0]	0(0.000)	[0]

Dictionary: MedDRA v16.0

◆ overlapping count
